# Applying the Findings of Public Health Research to Communities: Balancing Ideal Conditions With Real-World Circumstances

**Published:** 2008-03-15

**Authors:** Jaya K Rao

**Affiliations:** Centers for Disease Control and Prevention, Dr Rao is the Science Editor Fellow at *Preventing Chronic Disease*

**Figure 1:**
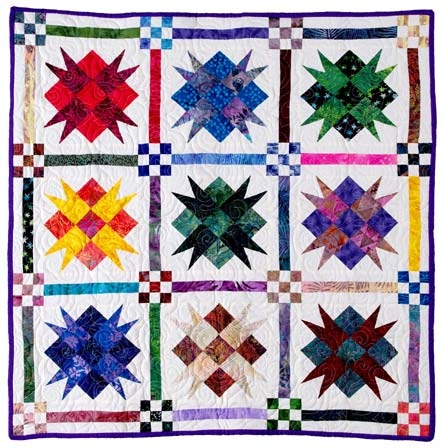
*Royal Star* quilt based on pattern by Debby Kratovil and pieced by the author. Photography by James Gathany, Centers for Disease Control and Prevention.

I have always been fascinated with quilts. The geometric patterns and vibrant colors of quilt tops remind me of the view through a kaleidoscope (Figure 1). As a quilter, I now realize that visually appealing optical illusions result from the balance of the colors, tones, and designs of fabrics within the quilt, and selecting and combining fabrics for a quilt is an art form itself. I find the boundless possibilities of fabric selection and combination the most exciting part of quilt making because I have the chance to imagine my finished quilt.

**Figure 2:**
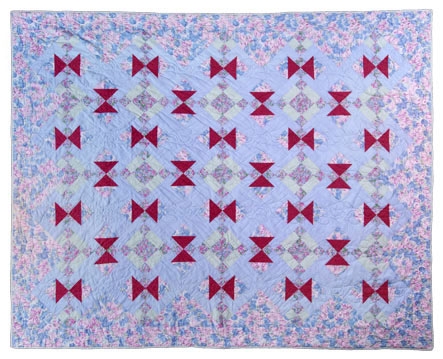
Hayes Corner quilt (pattern originator unknown) pieced by the author. Photography by James Gathany, Centers for Disease Control and Prevention.

I am not alone. All quilters use their imaginations when picking fabrics for quilt projects. Nowhere is this more evident than in the classes I take at a local quilt shop. Just seeing a sample quilt is usually enough to entice me to pay the registration fee. All students bring their fabrics to the first class, and we begin the same way, ready to receive instructions in cutting and sewing the pattern from the teacher. By the end of the last class, however, our different visions become apparent as each person holds up his or her quilt: light blues and lavenders peppered with bold maroon geometrics (Figure 2); striking African block prints in shades of black, brown, and ivory; simple cotton prints in baby pastels; or any other combination imaginable. The quilts are united by a common pattern, but each is unique and beautiful in its own way.

Quilts celebrate the myriad choices of individual quilters applied to the uniform elements of a quilt pattern. At the same time, we understand that our choices need balance — that large, bold prints can overwhelm delicate quilt patterns and that Christmas colors are not appropriate for a patriotically themed quilt. Applying the findings of public health research to the needs of communities requires similar attention to balance, but in public health, the balance is struck between idealized study conditions and real-world circumstances. This balance is the key to understanding the concepts of internal validity and external validity in research design. With the CONSORT (Consolidated Standards of Reporting Trials) criteria (1), editors and reviewers encourage authors to provide the details needed to assess the internal validity of their work. Procedures for allocating participants, strategies for minimizing study biases, and approaches for analyzing data to account for dropouts — these elements help readers understand the extent to which the observed findings are caused by the intervention rather than by extraneous factors.

**Figure 3:**
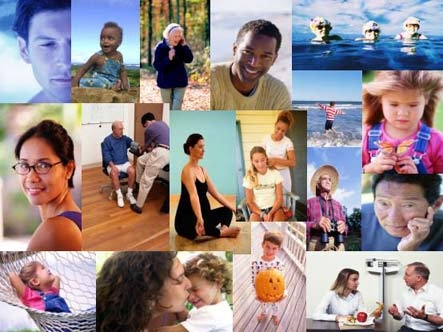
Information on study populations, settings, and interventions helps readers understand whether the findings are applicable to real communities. Photographs pieced by the author.

Although efficacy garners attention, a public health focus requires us to understand how these interventions apply to communities. Shadish et al defined external validity as "inferences about whether a causal relationship holds over variations in persons, settings, treatments and outcomes" (2). Ideally, research is conducted under tightly controlled conditions; for this reason, the settings, populations, and intervention components that comprise a study often differ from those of the community (3,4). Detailed information on intervention components and community characteristics would help readers understand whether study findings are generalizable to other settings and populations (Figure 3). Unfortunately, few articles include these details (3-5).

In a recent review of 119 health promotion interventions, Glasgow and colleagues found that few authors provided the information necessary for assessing the representativeness of their study settings and populations (6). For example, only 14% of articles included information on how well the study participants represented the target population, and only 16% described the participation rate at the level of study settings (6). Furthermore, authors provided variable amounts of information on study context and resources (e.g., costs, time) required for delivering the intervention (6). My colleagues and I reviewed 36 randomized controlled trials of interventions designed to improve physician–patient communication and found limited information on intervention characteristics (7). Among the interventions involving groups of participants, few authors described the facilitator–participant ratio, the number of contact hours per session, the frequency with which the intervention components were delivered, or the intervals between components (7). This lack of information on study settings, context, populations, and delivery of interventions in published reports limits the conclusions that systematic reviewers can make and hinders our ability to translate effective interventions into practice (6).

As helpful as the 22 CONSORT criteria are for assessing internal validity, they provide little guidance to authors on how to report issues of external validity (8): only one addresses generalizability. In April 2006, 12 editors of public health and health promotion journals, including *Preventing Chronic Disease*, met to discuss potential strategies for encouraging authors to include items related to external validity in their papers. A detailed summary of this meeting is available at http://www.re-aim.org (9). The following areas were identified by meeting participants as important aspects of external validity that should be reported (10):

1. Recruitment and selection procedures, participation rates, and representativeness of study participants, intervention staff, and delivery settings.
2. Level and consistency of implementation among program components, settings, staff, and time.
3. Effect on a variety of outcomes (11), especially outcomes important to populations, practitioners, and decision makers (e.g., quality of life, program costs, adverse consequences).
4. For follow-up reports, information on the rate of attrition at all levels (i.e., study participants, intervention staff, and delivery settings), long-term effects on outcomes, and program institutionalization, modification, or termination.

Many of these elements are already part of *Preventing Chronic Disease*'s guidance to authors of community case studies. This journal also endorses increased reporting on external validity in original research reports. To support such reporting, we

1. Encourage all authors submitting manuscripts to report on the recommended items related to external validity,
2. Ask reviewers to consider external validity when critiquing manuscripts,
3. Encourage the submission of articles that exemplify complete and thorough reporting on generalizability and external validity.

Experienced quilters can examine a pattern, visualize an array of fabrics that will result in an appealing quilt, and recognize the effort required to complete the work. Likewise, decision makers should be able to review reports of public health interventions and understand how these interventions might operate in their own community. Information on factors such as resource requirements, participation rates, and program sustainability are essential to help readers understand the applicability of public health interventions to their communities. By improving our reporting of external validity, public health practitioners and researchers will benefit as will the people they serve.
